# The Main Irish Grievance—Economic

**Published:** 1917-09-01

**Authors:** 


### NURSING AFFAIRS IN IRELAND.

### The Main Irish Grievance—Economic.

THE holiday season, such as it is in war-time, is drawing to a close, and in the near future many questions affecting the nursing profession will receive serious consideration. In Ireland, as you are aware, there is much pioneering to be done, and we have in our midst a new and very promising branch of the College, as well as a rival camp, each naturally endeavouring to strengthen its forces by recruiting. May I be permitted to offer a few remarks on the situation as it appears to an impartial observer: not from the standpoint of either institution, but rather from that of the working nurse who has entered the profession for a career. I venture to submit that the main grievance of the Irish nurse is the economic one, and it appears to me that this fails to receive adequate consideration. In other words, the pay and outlook of a trained nurse in Ireland are altogether out of proportion to her attainments, her responsibilities, and her hours of labour. Since the war broke out the contrast has become much more marked, and is likely to increase rather than to diminish. A very large number of nurses are quite apathetic as regards either the College or the opposition shop, and, so far as I am able to appreciate the cause, the reason is that they cannot discern any clear indication that their prospects and conditions of service are likely to receive sufficient consideration. Moreover, I believe that they will continue to occupy the fence until the outlook becomes more clearly defined. This impression may be erroneous, but, inasmuch as it has been conveyed to me by members of the rank and file, I venture to suggest that it is worth consideration.

### PARLOUS POSITION OF IRISH PROBATIONERS.

In Ireland the probationer, as I have already pointed out, works without remuneration for three years, commencing at the average age of two- or three-and-twenty. She then begins in a hospital at a salary of about £20 or £25, and after serving for a number of years with fidelity she may become a sister with a salary of from £30 to £40 or thereabouts. Her highest ambition is to become a matron, but the larger establishments usually employ matrons who have been trained in Great Britain, so that this possibility is, to say the least, comparatively remote. There are no pensions, no holiday-homes, no travelling facilities. In view of the extraordinary development of women's work in various directions, it is becoming every day more manifest that the profession of nursing is comparatively unattractive. The domestic servant, the woman munition-worker, the private secretary, the woman clerk, the school teacher—all of these have in recent times found an improving market for their services.

### WHY STATE REGISTRATION OFFERS NO ATTRACTIONS.

The Irish nurse remains, as they say in the Army, "as you were"—and, although State Registration may be within measurable distance of attainment, it must be obvious that it cannot by any possibility provide a remedy. It may, and certainly will, help to some extent, but its powers are distinctly limited, and for the elementary reason that it will not and cannot rid the market of the untrained worker. Every trained nurse owes it to herself and to her profession to do all in her power to speed up the necessary legislation. But the thoughtful nurse realises that no Act of Parliament which merely registers training, and which fails to create a monopoly thereby, can affect the pay and conditions of service of any class of workers. Other and more potent forces dominate the employment market.

### THE COLLEGE AND A POLICY OF ECONOMIC REFORM?

It is on this account that I suggest that the College of Nursing ought to open its campaign by some definite and authoritative policy of economic reform, because on such will depend its immediate and ultimate success. I entertain no doubt that this is included in the programme of the College propaganda, but, in Ireland especially, where the shoe pinches in an exceptional way, such an essential feature ought to occupy the foreground. Ask an intelligent and thinking nurse in Ireland her views as to the College programme, and it is more than probable that they will be found to be nebulous. Hence the apathy of the Irish rank and file, an apathy which will disappear like a fog before the rising sun when they realise that membership and betterment are synonymous terms.

### PAYMENT BY PLATITUDE.

As an ardent believer in the potentialities of the College, in its educational functions, in its wide outlook, I venture to suggest that an emphatic pronouncement on the economic question is not only desirable but imperative. Hitherto the nurse has been rewarded by public appreciation, but public appreciation unaccompanied by material benefit becomes a little wearying. For valuable public services payment by platitude is insufficient remuneration.

### A REMARKABLE TRIBUTE TO THE COLLEGE OF NURSING.

The Irish Nursing Board (the opposition shop referred to) has issued its bye-laws, accompanied by a circular-letter from Miss Carson Rae, the hon. secretary. Miss Rae, I may mention, is a Scottish lady trained in Westminster Hospital, and in this letter, doubtless unwittingly, she pays a remarkable tribute to the College of Nursing. "WE HOPE," she observes, "THAT THE IRISH NURSING BOARD WILL TAKE THE PLACE IN THIS COUNTRY THAT THE COLLEGE OF NURSING AIMS AT TAKING IN ENGLAND." This, from an out-and-out partisan of Mrs. Bedford Fenwick, takes one's breath away. And yet it demonstrates what I have so frequently iterated, that until the College established itself as an Imperial institution, and threatened to gather within its fold the whole of the British nursing profession, the Irish Nursing Association, of which the Irish Nursing Board is a development, never blinked an eye for the nurses save in the matter of extending moral support to the movement led by Mrs. Fenwick. Now, it is calmly proposed to ignore the fact that we have in our midst an Irish branch of the College of Nursing doing most successful propaganda work, and to adopt its functions. But I doubt if the College of Nursing has so far received a finer testimonial. There is, however, an essential difference between the potentialities of the two institutions. One (the College of Nursing) is Imperial, all-embracing; the other (the Irish Nursing Board) makes for isolation, and its policy, like that of Sinn Fein, is for "ourselves alone."

I hope to discuss the bye-laws in detail next week.—
Yours very truly, IERNE.

